# Supplementary material for: External validation of VO2max prediction models based on recreational and elite endurance athletes
Source: PLoS One. 2023 Jan 25;18(1):e0280897. doi: 10.1371/journal.pone.0280897 (PMC9876283; doi:10.1371/journal.pone.0280897)
Supplement: S1 File — (DOCX) [file pone.0280897.s001.docx]

Cardiopulmonary exercise testing protocol

Cardiopulmonary exercise tests (CPET) were preceded by body mass (BM) and fat mass (FM) analysis with 5 kHz/50 kHz/250 kHz electrical bioimpedance method on the body composition (BC) monitor (Tanita, MC 718, Japan. Conditions during BC and CPET were: 40 m^2^ indoor, air-conditioned area, 40–60% humidity, temperature 20–22°C, altitude 100 m MSL. Endurance athletes (EA) were instructed via e-mail on how to prepare: avoid any demanding exercises 24 hours before CPET, consume a high carbohydrate meal and hydrate with isotonic beverages 2-3 hours earlier, and exclude any stimulants or caffeine on the day of the procedure.

Cycle ergometry (CE) examination was performed on a cycle ergometry Cyclus-2 (RBM elektronik-automation GmbH, Leipzig, Germany) and treadmill (TE) examination was conducted on a mechanical treadmill (h/p/Cosmos quasar, Germany). CPET scores were measured breath by breath during 10-s intervals using a Hans Rudolph V2 Mask (Hans Rudolph, Inc, Shawnee, KS, USA), a gas exchange analyzer Cosmed Quark CPET (Rome, Italy), and dedicated manufacturer’s software (from PFT Suite to Omnia 10.0E.) HR was measured via ANT and a torso strap as a part of the Cosmed Quark set (product accuracy comparable to ECG; ± 1 bpm.). The CPET device was calibrated with reference gas (16% O_2_; 5% CO_2_) and turbine flow for each person separately, according to manufacturer recommendations. Equipment software was regularly actualized between 2013-2021. Three gas analyzing devices were utilized and each one has been changed after 36-48 months. Every part of CPET equipment was periodically verified by manufacturer employees to keep their mechanical certificates valid. Blood lactate (LA) was assessed with the usage of Super GL2 analyzer (Müller Gerätebau GmbH, Freital, Germany). The instrument was also individually prepared before each round of analysis and calibrated with reference solution before each sample set.

Exercises begin with a 5-min. warm-up (walking or pedaling with minimal resistance). Participants' endurance capacities were used to assess starting load. The initial power for CE was 60-150W and was increased in 2 min. intervals by 20-30W. The initial speed for TE was 7-12 km·h^-1^ (described by a person as a “conversation pace”) at 1% inclination. The pace was raised by 1 km·h^-1^ every 2 min. Observer verbally encouraged athletes to keep effort as long as possible due to assess their endurance most exactly. Achievement of oxygen uptake (VO_2_) or heart rate (HR) plateau, or volitional inability to maintain intensity were reasons for test termination. LA was measured by taking a 20 µL blood sample from a fingertip: directly prior to exercises, after any resistance or pace modification, and 3 min. after termination. Samples were obtained without an interruption in CE and TE tests. Before a proper sample was obtained, the first drops were gathered in a swab. HR (not averaged) was recorded at the highest point during intervals and used in further analysis^31^. Maximal oxygen uptake (VO_2max_) was defined as an averaged maximum oxygen uptake during the 15-s period at the end of the CPET.
