# Supplementary material for: External validation of VO2max prediction models based on recreational and elite endurance athletes
Source: PLoS One. 2023 Jan 25;18(1):e0280897. doi: 10.1371/journal.pone.0280897 (PMC9876283; doi:10.1371/journal.pone.0280897)
Supplement: S2 File — (DOCX) [file pone.0280897.s002.docx]

| Target variable (primary derived unit) | Reference | Target sex | Group size [M/F] | Age | Sample population characteristic and study type | Testing modality | Protocol | Prediction equation | R^2^ | Methodological quality [sum of points] |
| --- | --- | --- | --- | --- | --- | --- | --- | --- | --- | --- |
| VO_2peak_ (mL·min^-1^·kg^-1^) | Fitzgerald et. al[1] | F | 4884 F | Sedentary- 40.5 ± 19.4  Active- 34.7 ± 17.2  Endurance trained- 33.1 ± 14.1 | NR† | CE/TE | NR† | 72.41 – 0.62 · (age) | NR† | NR† |
| VO_2peak_ (mL·min^-1^·kg^-1^) | Wilson et. al[2] | M | 13828 M | Sedentary- 43 ± 18  Active- 38 ± 17  Endurance trained- 47 ± 16 | NR† | CE/TE | NR† | 77.2 – 0.46 · (age) | NR† | NR† |
| VO_2max_ (mL·min^-1^) | Wasserman et. al.[3] | M | 228/144 | 29-74 | Population-based (general population- shipyard workers); retrospective | CE/TE | NR | (weight) · (50.72 – (0.372 · (age)) | NR | NR |
| VO_2max_ (mL·min^-1^) | Wasserman et. al.[3] | F | 228/144 | 29-74 | Population-based (general population- shipyard workers); retrospective | CE/TE | NR | ((weight) + 42.8) · (22.78 – (0.17 · (age))) | NR | NR |
| VO_2max_/VO_2peak_ (mL·min^-1^·kg^-1^) | Myers et al.[4] | M/F | 4601/3158 | 20-79 | Population-based; retrospective | TE | Personalized incremental ramp protocol | 79.9 – 0.39 · (age) – 13.7 · (sex; M=0, F=1) – 0.127 · (body mass (lbs)) | 0.62 | 9 |
| VO_2max_/VO_2peak_ (mL·min^-1^·kg^-1^) | Kokkinos et al. (1)^§^[5] | M | 3378/1722 | 20-79 | Population-based; retrospective | CE | Personalized incremental ramp protocol | 1.74 · ((WR) ·6.12/(body mass)) + 3.5 | NR | 9 |
| VO_2max_/VO_2peak_ (mL·min^-1^·kg^-1^) | Kokkinos et al. (2)^§^[5] | F | 3378/1722 | 20-79 | Population-based; retrospective | CE | Personalized incremental ramp protocol | 1.76 × ((WR) · 6.12/(body mass)) + 3.5 | NR | 9 |
| VO_2max_/VO_2peak_ (mL·min^-1^·kg^-1^) | Kokkinos et al. (3)^§^[5] | M/F | 3378/1722 | 20-79 | Population-based; retrospective | CE | Personalized incremental ramp protocol | 1.65 · ((WR_peak_) ·6.12/(body mass)) + 3.5 | NR | 9 |
| VO_2peak_ (mL·min^-1^) | Mylius et al.[6] | M/F | 3570/907 | 7-65 | Population-based; retrospective | CE | Personalized incremental ramp protocol | −1469 + (673.00 · (sex; M= 1, F=0)) + (16.87 · (age)) + (−0.47 · (age)^2^) + (0.07 · (height)^2^) + (39.70 · (weight)) + (−0.16 × (weight)^2^) | 0.57 | 10 |
| VO_2max_  (mL·min^-1^·kg^-1^) | Nevill et al. (1)^§§^[7] | M/F | 4601/3158 | 46±13 | Population-based; retrospective | TE | Personalized incremental ramp protocol | (Body mass)^-0.854^ · (height)^1.44^ · exp · (0.424–0.346 · (sex; M=1, F=0) − 0.011 · (age)) | 0.65 | 9 |
| VO_2max_  (mL·min^-1^·kg^-1^) | Nevill et al. (2)^§§^[7] | M/F | 4601/3158 | 46±13 | Population-based; retrospective | TE | Personalized incremental ramp protocol | 41.38 – 10.88 · (sex) – 0.378 · (age) – 0.310 · (weight) + 0.227 · (height) | 0.63 | 9 |
| VO_2peak_ (L·min^-1^) | Petek et al.[8] | M/F | 189/83 | 45±15 | Endurance athletes; retrospective | TE | Incrimental ramp protocol; 0.5% grade/15 s | −0.83 · (sex) + 0.33 · (height) – 0.017 · (age) – 1.15 | 0.74 | 9 |
| VO_2peak_ (L·min^-1^) | Petek et al.[8] | M/F | 189/83 | 45±15 | Endurance athletes; retrospective | CE | Incrimental ramp protocol; 10-40 W/min | −0.72 · (sex) + 0.048 · (height) – 0.00019 · (age^2^) – 4.30 | 0.69 | 9 |

Table 1. Prediction equations included in validation. Abbreviations: M, male; F, female; TE, treadmill; CE, cycle ergometry; R^2^, coefficient of determination; VO_2max_; maximal oxygen uptake; VO_2peak_, oxygen uptake at peak exercise; WR, work rate (W). †Fitzgerald et al. and Wilson et al. are meta-analyses exclusively for one sex and one testing modality. ^§^Kokkinos et al. presents 3 equations for cycle ergometry: (1) only for males, (2) only for females, (3) for both males and females. ^§§^Nevill et al. presents 2 equations for treadmill: (1) allometric model, (2) additive linear model. Age is presented in years; height/ body height in cm; weight/body mass in kg (unless otherwise stated).

Table 1. Bibliography

1. Fitzgerald MD, Tanaka H, Tran Z v., Seals DR. Age-related declines in maximal aerobic capacity in regularly exercising vs. sedentary women: A meta-analysis. Journal of Applied Physiology. 1997;83.

2. Wilson TM, Tanaka H. Meta-analysis of the age-associated decline in maximal aerobic capacity in men: Relation to training status. American Journal of Physiology - Heart and Circulatory Physiology. 2000;278.

3. Wasserman K, Hansen JE, Sue DY, Stringer WW, Sietsema KE, Sun XG, et al. Principles of exercise testing and interpretation: Including pathophysiology and clinical applications: Fifth edition. Principles of Exercise Testing and Interpretation: Including Pathophysiology and Clinical Applications: Fifth Edition. 2011.

4. Myers J, Kaminsky LA, Lima R, Christle JW, Ashley E, Arena R. A Reference Equation for Normal Standards for VO2 Max: Analysis from the Fitness Registry and the Importance of Exercise National Database (FRIEND Registry). Progress in Cardiovascular Diseases. W.B. Saunders; 2017. p. 21–9.

5. Kokkinos P, Kaminsky LA, Arena R, Zhang J, Myers J. A new generalized cycle ergometry equation for predicting maximal oxygen uptake: The Fitness Registry and the Importance of Exercise National Database (FRIEND). European Journal of Preventive Cardiology. 2018;25.

6. Mylius CF, Krijnen WP, van der Schans CP, Takken T, Wittink H, Schmitz M, et al. Peak oxygen uptake reference values for cycle ergometry for the healthy dutch population: Data from the lowlands fitness registry. ERJ Open Research. 2019;5.

7. Nevill AM, Myers J, Kaminsky LA, Arena R. Improving reference equations for cardiorespiratory fitness using multiplicative allometric rather than additive linear models: Data from the Fitness Registry and the Importance of Exercise National Database Registry. Progress in Cardiovascular Diseases. W.B. Saunders; 2019. p. 515–21.

8. Petek BJ, Tso J v, Churchill TW, Guseh JS, Loomer G, DiCarli M, et al. Normative cardiopulmonary exercise data for endurance athletes: the C ardiopulmonary H ealth and E ndurance E xercise R egistry (CHEER) . European Journal of Preventive Cardiology. 2021;
