# Supplementary material for: External validation of VO2max prediction models based on recreational and elite endurance athletes
Source: PLoS One. 2023 Jan 25;18(1):e0280897. doi: 10.1371/journal.pone.0280897 (PMC9876283; doi:10.1371/journal.pone.0280897)
Supplement: S3 File — (DOCX) [file pone.0280897.s003.docx]

|  | Males | | | | | | Females | | | | | |
| --- | --- | --- | --- | --- | --- | --- | --- | --- | --- | --- | --- | --- |
|  | **TE [n=3330]** | | | **CE [n=1129]** | | | **TE [n=671]** | | | **CE [n=130]** | | |
| Variable | **Mean** | **CI** | **SD** | **Mean** | **CI** | **SD** | **Mean** | **CI** | **SD** | **Mean** | **CI** | **SD** |
| Age | 35.90 | 35.62-36.18 | 8.15 | 37.28 | 36.74-37.82 | 9.26 | 33.86 | 33.26-34.43 | 7.74 | 33.17 | 31.88-34.46 | 7.43 |
| VO_2AT_ | 38.42^*#^ | 38.25-38.59 | 4.96 | 33.12 | 32.78-33.46 | 5.81 | 35.69^#^ | 35.32-36.05 | 4.83 | 32.71 | 31.84-33.58 | 4.99 |
| VO_2ATA_ | 2955.15^*#^ | 2942.04-2968.26 | 385.79 | 2579.54^*^ | 2556.40-2602.68 | 396.27 | 2137.77^#^ | 2113.25-2162.29 | 323.48 | 1994.46 | 1939.91-2049.00 | 314.32 |
| RER_AT_ | 0.87^*^ | 0.87-0.87 | 0.04 | 0.87 | 0.87-0.87 | 0.04 | 0.86 | 0.85-0.86 | 0.04 | 0.87 | 0.86-0.87 | 0.04 |
| HR_AT_ | 151.32^*#^ | 150.96-151.68 | 10.70 | 141.80^*^ | 141.12-142.47 | 11.58 | 156.45^#^ | 155.66-157.24 | 10.39 | 148.52 | 146.41-150.64 | 12.18 |
| VE_AT_ | 78.26^*#^ | 77.84-78.68 | 12.02 | 65.42^*^ | 64.77-66.07 | 10.97 | 58.38^#^ | 57.66-59.09 | 9.25 | 52.06 | 50.54-53.57 | 8.53 |
| fR_AT_ | 34.88^#^ | 34.61-35.14 | 7.85 | 27.51^*^ | 27.19-27.83 | 5.47 | 34.89^#^ | 34.31-35.47 | 7.66 | 28.45 | 27.57-29.34 | 5.10 |
| LA_AT_ | 1.95^#^ | 1.92-1.98 | 0.67 | 1.84 | 1.81-1.88 | 0.58 | 1.86 | 1.80-1.93 | 0.66 | 1.75 | 1.63-1.86 | 0.57 |
| VO_2RCP_ | 47.59^*#^ | 47.37-47.81 | 6.10 | 44.44 | 43.99-44.89 | 7.41 | 43.05 | 42.56-43.55 | 6.14 | 42.45 | 41.30-43.60 | 6.20 |
| VO_2RCPA_ | 3642.72^*#^ | 3626.90-3658.54 | 465.70 | 3446.11^*^ | 3417.06-3475.15 | 497.40 | 2576.01 | 2545.15-2606.87 | 407.12 | 2571.40 | 2500.83-2641.96 | 406.63 |
| RER_RCP_ | 1.00^*#^ | 1.00-1.00 | 0.04 | 1.01 | 1.01-1.01 | 0.04 | 0.99^#^ | 0.99-1.00 | 0.04 | 1.01 | 1.00-1.01 | 0.03 |
| HR_RCP_ | 173.43^*#^ | 173.12-173.75 | 9.33 | 168.61^*^ | 168.00-169.21 | 10.41 | 176.04^#^ | 175.34-176.73 | 9.12 | 172.43 | 170.68-174.19 | 10.11 |
| VE_RCP_ | 113.82^*#^ | 113.25-114.39 | 16.43 | 107.30^*^ | 106.25-108.36 | 17.81 | 81.15 | 80.20-82.11 | 12.34 | 79.12 | 76.69-81.54 | 13.64 |
| fR_RCP_ | 44.19^#^ | 43.91-44.48 | 8.52 | 37.90 | 37.48-38.32 | 7.16 | 43.09^#^ | 42.49-43.68 | 7.87 | 37.51 | 36.46-38.56 | 6.05 |
| LA_RCP_ | 4.53^*#^ | 4.49-4.58 | 1.07 | 5.05 | 4.97-5.13 | 1.15 | 4.19^#^ | 4.09-4.29 | 1.02 | 4.87 | 4.66-5.09 | 1.06 |
| VO_2max_ | 54.10^*#^ | 53.87-54.34 | 6.93 | 51.92^*^ | 51.45-52.39 | 8.05 | 48.73 | 48.23-49.24 | 6.67 | 49.05 | 47.90-50.20 | 6.64 |
| VO_2maxA_ | 4176.37^*#^ | 4157.64-4195.09 | 551.09 | 4058.88^*^ | 4026.35-4091.41 | 557.09 | 2949.02 | 2911.51-2986.54 | 494.89 | 3003.35 | 2921.46-3085.24 | 471.91 |
| RER_max_ | 1.12^#^ | 1.12-1.12 | 0.04 | 1.13 | 1.13-1.13 | 0.05 | 1.12 | 1.12-1.12 | 0.04 | 1.12 | 1.12-1.13 | 0.03 |
| HR_max_ | 184.81^#^ | 184.49-185.13 | 9.54 | 182.86 | 182.27-183.44 | 9.97 | 185.39 | 184.69-186.09 | 9.24 | 184.73 | 183.04-186.43 | 9.77 |
| VE_max_ | 148.86^*#^ | 148.15-149.57 | 20.46 | 158.55^*^ | 157.04-160.06 | 25.45 | 103.83^#^ | 102.60-105.05 | 15.86 | 113.63 | 110.25-117.01 | 19.00 |
| fR_max_ | 57.59^#^ | 57.28-57.90 | 9.20 | 56.50 | 55.95-57.05 | 9.36 | 55.46 | 54.83-56.09 | 8.30 | 55.29 | 53.93-56.65 | 7.86 |
| LA_max_ | 9.91^*#^ | 9.82-10.00 | 2.02 | 11.37 | 11.21-11.54 | 2.36 | 9.08^#^ | 8.88-9.28 | 1.93 | 10.86 | 10.37-11.35 | 2.32 |
| S_ATA_/P_ATA_^§^ | 10.97^*^ | 10.92-11.02 | 1.40 | 178.70^*^ | 176.75-180.66 | 33.48 | 9.64 | 9.53-9.74 | 1.36 | 139.41 | 135.06-143.76 | 25.05 |
| S_RCPA_/P_RCPA_^§^ | 14.02^*^ | 13.96-14.08 | 1.74 | 261.84^*^ | 259.32-264.36 | 43.18 | 12.29 | 12.16-12.41 | 1.68 | 198.84 | 192.98-204.69 | 33.76 |
| S_maxA_/P_maxA_^§^ | 16.07^*^ | 16.01-16.14 | 1.93 | 315.31^*^ | 312.42-318.20 | 49.46 | 14.12 | 13.98-14.26 | 1.85 | 239.14 | 232.56-245.73 | 1.85 |
| P_AT_^§^ | n/a | n/a | n/a | 2.41 | 2.38-2.45 | 0.50 | n/a | n/a | n/a | 2.40 | 2.31-2.49 | 0.48 |
| P_RCP_^§^ | n/a | n/a | n/a | 3.47 | 3.43-3.52 | 0.66 | n/a | n/a | n/a | 3.37 | 3.25-3.48 | 0.60 |
| P_max_^§^ | n/a | n/a | n/a | 4.19 | 4.14-4.24 | 0.75 | n/a | n/a | n/a | 4.07 | 3.94-4.20 | 0.66 |
| S_S_/P_S_^§^ | 8.61 | 8.56-8.66 | 1.28 | 102.82 | 101.04-104.59 | 27.64 | 7.60 | 7.51-7.69 | 1.08 | 85.39 | 81.59-89.19 | 19.35 |

Table 1. CPET characteristics. Relative values for speed on TE were not calculated. Abbreviations: CI, confidence interval; SD, standard deviation; VO_2AT_, relative VO_2_ at AT (mL·min^-1^·kg^-1^); VO_2ATA_, absolute VO_2_ at AT (mL·min^-1^); RER_AT_, respiratory exchange ratio at AT; HR_AT_, heart rate at AT (bpm); VE_AT_, pulmonary ventilation at AT (L·min^-1^); fR_AT_, respiratory frequency at AT (breaths per minute); LA_AT_, lactate concentration at AT (mmol·L^-1^); VO_2RCP_, relative VO_2_ at RCP (mL·min^-1^·kg^-1^); VO_2RCPA_, absolute VO_2_ at RCP (mL·min^-1^); VO_2RCP_, relative VO_2_ at RCP (mL·min^-1^·kg^-1^); RER_RCP_, respiratory exchange ratio at RCP; HR_RCP_, heart rate at RCP (bpm); VE_RCP_, pulmonary ventilation at RCP (L·min^-1^); fR_RCP_, respiratory frequency at RCP (breaths per minute); LA_max_, lactate concentration at RCP (mmol·L^-1^); VO_2max_, relative maximal VO_2_ (mL·min^-1^·kg^-1^); VO_2maxA_, absolute maximal VO_2_ (mL·min^-1^); RER_max_, maximal respiratory exchange ratio; HR_max_, maximal heart rate (bpm); VE_max_, maximal pulmonary ventilation (L·min^-1^); fR_max_, maximal respiratory frequency; LA_max_, maximal lactate concentration (mmol·L^-1^); S_ATA_, absolute speed at AT (km·h^-1^); P_ATA_, absolute power at AT (watt); S_RCPA_, absolute speed at RCP (km·h^-1^); P_RCPA_, absolute power at RCP (watt); S_maxA_, maximal absolute speed (km·h^-1^); P_maxA_, maximal absolute power (watt); P_AT_, relative power at AT (watt· body mass in kg^-1^); n/a, not applicable; P_RCP_, relative power at RCP (watt· body mass in kg^-1^); P_max_, relative maximal power (watt· body mass in kg^-1^); S_S_, absolute protocol starting speed (km·h^-1^); P_S_, absolute protocol starting power (watt). Age is presented in years. Number of lactate concentration (LA) tests were: 833 for males/CE, 2140 for males/TE, 96 for females/CE and 405 for females/TE (not all participants decided for LA test because it was an optional variable in clinic’s exertion tests portfolio). Comparisons between subgroups (*p* value) were obtained by one-way ANOVA and post-hoc HSD Tukey test. Significant values (*p<0.05*) were marked as [^*^] for differences between males and females for TE/CE, and [^#^] for differences between TE and CE for males or females. ^§^speed for TE, power for CE.
