# Supplementary material for: External validation of VO2max prediction models based on recreational and elite endurance athletes
Source: PLoS One. 2023 Jan 25;18(1):e0280897. doi: 10.1371/journal.pone.0280897 (PMC9876283; doi:10.1371/journal.pone.0280897)
Supplement: S4 File — (DOCX) [file pone.0280897.s004.docx]

|  | Males | | | Females | | |
| --- | --- | --- | --- | --- | --- | --- |
| Prior Equations in CE | | | | | | |
| Validated subgroup | **HTEA‡**  **[n=69]** | **REA**  **[n=429]** | **LTEA‡**  **[n=81]** | **HTEA‡**  **[n=10]** | **REA‡**  **[n=57]** | **LTEA‡**  **[n=11]** |
| Actual V̇O_2max_ in validated subgroup for CE | 62.11^BC^ (7.50) | 51.63^AC^ (5.43) | 40.18^AB^ (4.91) | 56.77^BC^ (3.43) | 49.03^AC^ (4.39) | 40.26^AB^ (6.61) |
| P_RCP_^¶^ in validated subgroup for CE | 4.10  (0.53) | 3.53  (0.47) | 2.47  (0.54) | 4.23  (1.18) | 3.45  (0.48) | 2.53  (0.32) |
| Wilson et al.† | 60.29 (4.81) | 59.70^*^ (3.94) | 60.20^*^  (4.27) | n/a | n/a | n/a |
| Fitzgerald et al. † | n/a | n/a | n/a | 54.20  (4.33) | 53.58^*^ (5.19) | 54.91^*^  (4.52) |
| Wasserman et al. | 37.05^*^  (3.89) | 36.57 (3.19) | 36.97^*^  (3.45) | 30.21^*^  (2.28) | 29.06^*^  (2.95) | 28.98^*^  (3.35) |
| Kokkinos et al. (1 for males/ 2 for females)^§^ | 59.49^*BC^ (5.19) | 44.08^*AC^ (3.19) | 31.76^*AB^ (4.35) | 56.55^BC^ (6.54) | 44.70^*AC^ (6.43) | 35.02^*AB^ (4.04) |
| Kokkinos et al. (3)^§^ | 56.60^*BC^ (4.92) | 46.29^*AC^ (3.36) | 30.28^*AB^ (4.13) | 53.24^BC^ (6.13) | 42.12^*AC^ (6.03) | 33.05^*AB^ (3.79) |
| Mylius et al. | 42.09^BC^  (4.23) | 38.85^*AC^  (3.86) | 35.40^*AB^  (5.68) | 33.29^*^  (2.77) | 32.29^*^  (3.22) | 35.3^*^9  (5.68) |
| Petek et al. | 57.12^*BC^  (5.43) | 51.57^*AC^  (5.22) | 46.19^*AB^ (7.91) | 48.43^*^ (4.04) | 46.27^*^  (4.83) | 45.33  (7.86) |
| Prior Equations in TE | | | | | | |
| Validated subgroup | **HTEA**  **[n=188]** | **REA**  **[n=1282]** | **LTEA**  **[n=209]** | **HTEA‡**  **[n=49]** | **REA**  **[n=241]** | **LTEA‡**  **[n=44]** |
| Actual V̇O_2max_ in validated subgroup for TE | 64.31^BC^ (6.03) | 52.92^AC^ (5.41) | 45.65^AB^ (5.53) | 58.29^BC^ (5.81) | 49.20^AC^ (4.74) | 40.15^AB^ (4.61) |
| S_RCP_^¶^ in validated subgroup for TE | 17.45 (0.91) | 14.05 (0.66) | 10.58  (1.02) | 15.40  (1.06) | 12.27 (0.66) | 9.24  (0.59) |
| Wilson et al. † | 61.73^*BC^ (3.69) | 60.59^*A^ (3.86) | 60.24^*A^ (4.06) | n/a | n/a | n/a |
| Fitzgerald et al.† | n/a | n/a | n/a | 54.19^*^ (4.91) | 53.43^*^ (4.88) | 52.89^*^ (5.10) |
| Wasserman et al. | 38.21^*BC^  (2.90) | 37.28^*A^ (3.12) | 37.01^*A^  (3.29) | 30.14^*C^  (3.34) | 29.16^*^  (2.68) | 28.14^*A^  (3.22) |
| Myers et al. | 46.84^*BC^ (3.89) | 44.10^*AC^ (4.15) | 41.88^*AB^ (5.00) | 36.92^*C^ (4.72) | 35.90^*C^ (3.73) | 34.23^*AB^ (4.85) |
| Nevill et al. (1)^§§^ | 48.77^*BC^ (5.79) | 44.63^*AC^ (5.78) | 41.53^*AB^ (6.64) | 38.23^*BC^ (5.15) | 35.93^*A^ (4.37) | 34.16^*A^ (6.47) |
| Nevill et al. (2)^§§^ | 47.09^*BC^ (3.78) | 44.46^*AC^ (4.07) | 42.03^*AB^ (5.14) | 38.19^*C^ (4.04) | 36.79^*^ (3.65) | 35.27^*A^ (5.34) |
| Petek et al. | 58.95^*BC^  (5.37) | 54.18^AC^  (5.49) | 50.11^*AB^  (7.25) | 52.35^*BC^  (6.15) | 49.13^AC^  (5.24) | 46.62^*AB^  (8.85) |

Table 1. Comparison between observed and predicted VO_2max_ from models selected for validation stratified by endurance level and sex. Abbreviations: CE, cycle ergometry; HTEA, high-trained endurance athletes; REA, recreational. endurance athletes; LTEA, low-trained endurance athletes; V̇O_2max_, maximal oxygen uptake; n/a, not applicable; TE, treadmill. Continuous value is presented as mean (SD). Values are presented in mL·kg^-1^·min^-1^. Comparisons between subgroups (*p* value) were obtained by one-way ANOVA, Student’s t-test, and post-hoc HSD Tukey test. Significant values (*p<0.05*) were marked as [^*^] for differences between actual and predicted V̇O_2max_, and independently for differences between CE or TE for males and females as [^A^] for HTEA, [^B^] for REA, [^C^] for LTEA. †Fitzgerald et al. and Wilson et al. are meta-analyses exclusively for one sex. ^§^Kokkinos et al. presents 3 equations for cycle ergometry: (1) only for males, (2) only for females, (3) for both males and females. ^§§^Nevill et al. presents 2 equations for treadmill: (1) allometric model, (2) additive model. Subgroups that did not meet the TRIPOD guidelines to consider their validation results as reliable (i.e n≥100) were marked with [‡]. ^¶^P_RCP_, relative power at RCP (watt· body mass in kg^-1^); and S_RCP_, absolute speed at RCP (km·h^-1^) are additionally presented in the table because based on these parameters, endurance group classification has been done.

| Prior Equations in CE | | | Prior Equations in TE | | |
| --- | --- | --- | --- | --- | --- |
| Validated subgroup | **Males**  **[n=1129]** | **Females**  **[n=130]** |  | **Males**  **[n=3330]** | **Females**  **[n=671]** |
| Actual V̇O_2max_ in validated subgroup for CE/TE | 51.92 (8.05) | 49.04  (6.66) |  | 54.10 (6.93) | 48.73  (6.67) |
| Wilson et al.† | 60.07^*^ (4.19) | n/a | **Wilson et al. †** | 60.69^*^ (3.75) | n/a |
| Fitzgerald et al. † | n/a | 53.85^*^ (4.61) | **Fitzgerald et al.†** | n/a | 53.43^*^ (4.80) |
| Wasserman et al. | 36.87^*^ (3.39) | 29.27^*^ (2.87) | **Wasserman et al.** | 37.37^*^ (3.03) | 29.22^*^ (2.89) |
| Kokkinos et al. (1 for males/ 2 for females)^§^ | 46.51^*^ (7.80) | 45.36^*^ (7.82) | **Myers et al.** | 44.14^*^ (4.23) | 35.96^*^ (4.01) |
| Kokkinos et al. (3)^§^ | 44.29^*^ (7.39) | 42.74^*^ (7.33) | **Nevill et al. (1)^§§^** | 44.71^*^ (5.97) | 36.02^*^ (4.84) |
| Mylius et al. | 39.13^*^ (4.56) | 32.42^*^ (3.16) | **Nevill et al. (2)^§§^** | 44.48^*^ (4.21) | 36.78^*^ (3.94) |
| Petek et al. | 52.00 (6.30) | 46.39^*^ (5.53) | **Petek et al.** | 54.21 (5.99) | 49.25  (5.97) |

Table 2. Comparison between observed and predicted VO_2max_ from models selected for validation calculated for whole population and stratified by endurance level and sex. Abbreviations: CE, cycle ergometry; V̇O_2max_, maximal oxygen uptake; n/a, not applicable; TE, treadmill. Continuous value is presented as mean (SD). Values are presented in mL·kg^-1^·min^-1^. Comparisons between subgroups (*p* value) were obtained by one-way ANOVA, Student’s t-test, and post-hoc HSD Tukey test. Significant values (*p<0.05*) were marked as [^*^] for differences between actual and predicted V̇O_2max_. For equations primarily derived for both sexes, values are presented for whole group, while for equations derived exclusively for males and females, results are presented for one sex. †Fitzgerald et al. and Wilson et al. are meta-analyses exclusively for one sex. ^§^Kokkinos et al. presents 3 equations for cycle ergometry: (1) only for males, (2) only for females, (3) for both males and females. ^§§^Nevill et al. presents 2 equations for treadmill: (1) allometric model, (2) additive model. Subgroups that did not meet the TRIPOD guidelines to consider their validation results as reliable (i.e n≥100) were marked with [‡].

|  | Males | | | | Females | | | |
| --- | --- | --- | --- | --- | --- | --- | --- | --- |
| Prior Equations in CE | | | | | | | | |
| Validated subgroup | **Age**  **18-30**  **[n=228]** | **Age**  **31-45**  **[n=733]** | **Age**  **46-60**  **[n=154]** | **Age**  **≥61‡**  **[n=14]** | **Age**  **18-30‡**  **[n=48]** | **Age**  **31-45‡**  **[n=77]** | **Age**  **46-60‡**  **[n=5]** | **Age ≥61‡**  **[n=0]** |
| Actual V̇O_2kmax_ in validated subgroup for CE | 56.88^BCD^  (8.66) | 51.35^ACD^ (7.29) | 48.03^ABD^ (6.94) | 43.28^ABCD^ (6.96) | 51.03^B^ (6.02) | 47.94^A^ (6.85) | 47.12 (5.21) | ­n/a |
| Wilson et al.† | 65.90^*BCD^ (1.88) | 59.78^*ACD^ (1.92) | 53.95^*ABD^ (1.73) | 47.56^*ABCD^ (1.47) | n/a | n/a | n/a | n/a |
| Fitzgerald et al.† | n/a | n/a | n/a | n/a | 58.56^*BC^ (2.38) | 51.54^*AC^ (2.55) | 44.03^AB^ (2.10) | n/a |
| Wasserman et al. | 41.58^*BCD^ (1.52) | 36.63^*ACD^ (1.56) | 31.91^*ABD^ (1.40) | 26.75^*ABCD^ (1.19) | 31.85^*BC^ (2.21) | 27.93^*AC^ (1.90) | 25.24^*AB^ (1.99) | n/a |
| Kokkinos et al.  (1 for males/ 2 for females)^§^ | 50.67^*BCD^ (8.57) | 46.03^*ACD^ (7.26) | 43.33^*ABD^ (6.55) | 39.12 ^ABCD^  (5.43) | 47.72^*B^ (9.44) | 44.11^*A^ (6.51) | 41.87 (2.62) | n/a |
| Kokkinos et al. (3)^§^ | 48.23^*BCD^ (8.12) | 43.83^*ACD^ (6.88) | 41.27^*ABD^ (6.21) | 37.28^*ABCD^ (5.15) | 44.96^*B^ (8.85) | 41.57^*A^ (6.10) | 39.47^*^ (2.45) | n/a |
| Mylius et al. | 43.56^*BCD^ (3.20) | 39.01^*ACD^(3.62) | 34.16^*ABD^  (2.95) | 27.93^*ABCD^  (2.97) | 34.66^*BC^  (2.14) | 31.41^*AC^ (2.67) | 22.82^*AB^ (2.95) | n/a |
| Petek et al. | 57.51^BCD^  (5.39) | 51.49^ACD^  (5.59) | 47.04^ABD^  (4.59) | 43.78^ABCD^  (4.59) | 49.31^BC^  (4.78) | 44.86^*AC^  (5.21) | 41.85^A^  (5.51) | n/a |
| Prior Equations in TE | | | | | | | | |
| Validated subgroup | **Age**  **18-30**  **[n=871]** | **Age**  **31-45**  **[n=2109]** | **Age**  **46-60**  **[n=333]** | **Age**  **≥61‡**  **[n=17]** | **Age**  **18-30**  **[n=233]** | **Age**  **31-45**  **[n=391]** | **Age**  **46-60‡**  **[n=46]** | **Age ≥61‡**  **[n=1]** |
| Actual V̇O_2kmax_ in validated subgroup for TE | 57.05^BCD^ (6.95) | 53.50^ACD^ (6.58) | 50.60^ABD^ (6.12) | 45.91^ABC^ (5.31) | 50.40^BC^ (6.60) | 48.22^AC^ (6.44) | 44.65^AB^ (6.66) | n/a |
| Wilson et al.† | 65.33^*BCD^ (1.61) | 59.88^*ACD^ (1.86) | 54.33^*ABD^ (1.56) | 47.81^BCD^ (1.68) | n/a | n/a | n/a | n/a |
| Fitzgerald et al.† | n/a | n/a | n/a | n/a | 58.58^*BC^ (2.22) | 51.51^*AC^ (2.35) | 43.98^AB^ (1.62) | n/a |
| Wasserman et al. | 41.12^*BCD^ (1.30) | 36.71^*ACD^ (1.50) | 32.22^*ABD^ (1.26) | 26.96^*ABC^ (1.36) | 31.93^*BC^ (1.90) | 26.18^*AC^ (1.97) | 24.60^*AB^ (1.47) | n/a |
| Myers et al. | 48.35^*BCD^ (3.07) | 43.32^*ACD^ (2.62) | 38.79^*ABD^ (2.74) | 34.28^*ABCD^ (2.62) | 39.62^*B^ (2.44) | 34.52^*AC^ (2.96) | 29.83^*AB^  (2.44) | n/a |
| Nevill et al. (1)^§§^ | 50.79^*BCD^ (5.10) | 43.39^*ACD^ (4.23) | 37.77^*ABD^ (3.38) | 32.89^*ABC^ (2.52) | 40.15^*BC^ (3.61) | 34.35^*AC^ (3.67) | 29.39^*AB^ (2.65) | n/a |
| Nevill et al. (2)^§§^ | 48.87^*BCD^ (2.99) | 43.63^*ACD^ (2.95) | 38.94^*ABD^ (2.61) | 34.15^*ABC^ (2.21) | 40.45^*BC^ (2.31) | 35.37^*AC^ (2.76) | 30.31^*AB^ (2.38) | n/a |
| Petek et al. | 57.69^*BCD^  (6.00) | 53.41^ACD^  (5.46) | 50.48^AB^  (4.88) | 48.36^AB^  (3.74) | 52.88^*BC^  (5.01) | 47.82^AC^  (5.45) | 43.09^AB^  (4.19) | n/a |

Table 3. Comparison between observed and predicted VO_2max_ from models selected for validation stratified by age and sex. Abbreviations: CE, cycle ergometry; V̇O_2max_, maximal oxygen uptake; n/a, not applicable; TE, treadmill. Continuous value is presented as mean (SD). Values are presented in mL·kg^-1^·min^-1^. Comparisons between subgroups (*p* value) were obtained by one-way ANOVA, Student’s t-test, and post-hoc HSD Tukey test. Significant values (*p<0.05*) were marked as [^*^] for differences between actual and predicted V̇O_2max_, and independently for differences between CE or TE for males and females as [^A^] for 18-30, [^B^] for 31-45, [^C^] for 46-60, [^D^] for ≥61. Due to the too small group size for females ≥61 years, the validation was not carried out. †Fitzgerald et al. and Wilson et al. are meta-analyses exclusively for one sex. ^§^Kokkinos et al. presents 3 equations for cycle ergometry: (1) only for males, (2) only for females, (3) for both males and females. ^§§^Nevill et al. presents 2 equations for treadmill: (1) allometric model, (2) additive model. Subgroups that did not meet the TRIPOD guidelines to consider their validation results as reliable (i.e n≥100) were marked with [‡].

| Prior equation in CE | | | | | | | | | | | | |
| --- | --- | --- | --- | --- | --- | --- | --- | --- | --- | --- | --- | --- |
| Validated subgroup | **All [n=1259]** | | | | **Males [n=1129]** | | | | **Females [n=130]** | | | |
|  | **R^2^** | **RMSE** | **C1** | **C2** | **R^2^** | **RMSE** | **C1** | **C2** | **R^2^** | **RMSE** | **C1** | **C2** |
| Wilson et al.† (mL·min^-1^·kg^-1^) | n/a | n/a | n/a | n/a | n/a | 6.86 | 15.20^*^ | 0.63^*^ | n/a | n/a | n/a | n/a |
| Fitzgerald et al.† (mL·min^-1^·kg^-1^) | n/a | n/a | n/a | n/a | n/a | n/a | n/a | n/a | n/a | n/a | n/a | n/a |
| Wasserman et al. (mL·min^-1^) | n/a | n/a | n/a | n/a | 0.26^*^ | 476.22 | 2153.47^*^ | 0.69^*^ | 0.30^*^ | 420.58 | 398.59^*^ | 1.45^*^ |
| Kokkinos et al. (1 for males/ 2 for females)^§^ (mL·min^-1^·kg^-1^) | n/a | n/a | n/a | n/a | 0.64^*^ | 4.76 | 13.13^*^ | 0.83^*^ | 0.65 | 4.75 | 16.31^*^ | 0.72^*^ |
| Kokkinos et al. (3)^§^ (mL·min^-1^·kg^-1^) | 0.64^*^ | 4.77 | 13.09^*^ | 0.87^*^ | 0.65^*^ | 4.75 | 12.97^*^ | 0.88^*^ | 0.55^*^ | 4.46 | 16.14^*^ | 0.76^*^ |
| Mylius et al. (mL·min^-1^) | 0.41^*^ | 495.94 | 1136.65^*^ | 0.95^*^ | 0.21^*^ | 495.10 | 1346.91^*^ | 0.89^*^ | 0.14^*^ | 500.47 | 1283.10^*^ | 0.85^*^ |
| Petek et al. (L·min^-1^) | 0.38^*^ | 0.51 | 0.87^*^ | 0.78^*^ | 0.19^*^ | 0.50 | 1.28^*^ | 0.68^*^ | 0.07^*^ | 0.52 | 1.51^*^ | 0.52^*^ |
| Prior equation in TE | | | | | | | | | | | | |
| Validated subgroup | **All [n=4001]** | | | | **Males [n=3330]** | | | | **Females [n=671]** | | | |
|  | **R^2^** | **RMSE** | **C1** | **C2** | **R^2^** | **RMSE** | **C1** | **C2** | **R^2^** | **RMSE** | **C1** | **C2** |
| Wilson et al.† (mL·min^-1^·kg^-1^) | n/a | n/a | n/a | n/a | 0.11^*^ | 6.86 | 15.20^*^ | 0.63^*^ | n/a | n/a | n/a | n/a |
| Fitzgerald et al.† (mL·min^-1^·kg^-1^) | n/a | n/a | n/a | n/a | n/a | n/a | n/a | n/a | 0.02 | 6.42 | 28.51^*^ | 0.38^*^ |
| Wasserman et al. (mL·min^-1^) | n/a | n/a | n/a | n/a | 0.26^*^ | 476.22 | 2153.47^*^ | 0.69^*^ | 0.30^*^ | 420.58 | 398.59^*^ | 1.45^*^ |
| Myers et al. (mL·min^-1^·kg^-1^) | 0.29^*^ | 6.03 | 21.28 | 0.75^*^ | 0.25^*^ | 5.99 | 17.87^*^ | 0.82^*^ | 0.15^*^ | 6.14 | 25.40^*^ | 0.65^*^ |
| Nevill et al. (1) ^§§^ (mL·min^-1^·kg^-1^) | 0.29^*^ | 6.04 | 28.06^*^ | 0.58^*^ | 0.24^*^ | 6.04 | 28.70^*^ | 0.57^*^ | 0.18* | 6.04 | 27.77^*^ | 0.58^*^ |
| Nevill et al. (2) ^§§^ (mL·min^-1^·kg^-1^) | 0.28^*^ | 6.05 | 20.49^*^ | 0.76^*^ | 0.24^*^ | 6.03 | 18.16^*^ | 081^*^ | 0.15^*^ | 6.13 | 24.37^*^ | 0.66^*^ |
| Petek et al. (L·min^-1^) | 0.54^*^ | 0.48 | 0.04 ^*^ | 0.99^*^ | 0.20^*^ | 0.48 | 0.32 ^*^ | 0.93^*^ | 0.25^*^ | 0.42 | 0.35^*^ | 0.88 ^*^ |

Table 4. Performance of selected models calculated for whole population stratified by endurance level and sex. Abbreviations: CE, cycle ergometry; R^2^, adjusted R^2^; RMSE, root mean square error; C1, calibration-in-the-large; C2, calibration slope; n/a, not applicable; TE, treadmill. Comparisons between subgroups (*p* value) were obtained by one-way ANOVA, Student’s t-test, and post-hoc HSD Tukey test. Significant values (*p<0.05*) were marked as [^*^]. All values are presented in originally derived units for each model (original unit was added in bracket). For equations primarily derived for both sexes, model’s performance is presented for whole group, while for equations derived exclusively for males and females, model’s performance is presented for one sex. †Fitzgerald et al. and Wilson et al. are meta-analyses exclusively for one sex. ^§^Kokkinos et al. presents 3 equations for cycle ergometry: (1) only for males, (2) only for females, (3) for both males and females. ^§§^Nevill et al. presents 2 equations for treadmill: (1) allometric model, (2) additive model. Subgroups that did not meet the TRIPOD guidelines to consider their validation results as reliable (i.e n≥100) were marked with [‡].
