# Supplementary material for: External validation of VO2max prediction models based on recreational and elite endurance athletes
Source: PLoS One. 2023 Jan 25;18(1):e0280897. doi: 10.1371/journal.pone.0280897 (PMC9876283; doi:10.1371/journal.pone.0280897)
Supplement: Attachment — Submitted filename: Response to Reviewers1.docx [file pone.0280897.s006.docx]

Author’s response to reviews

Dear reviewers,

We are grateful for the time and effort you took to write these extensive reviews. We have done our best to incorporate all of your suggestions and send back the revised manuscript. We believe the content is much improved and more clear for readers thanks to your comments.

Yours sinserely,

Szczepan Wiecha on behalf of all authors.

In the following, please find our answers to your comments.

Title: External validation of VO2max prediction models based on recreational and elite

endurance athletes

Version: 1 Date: 30 Aug 2022

In the following, please find our answers to your comments.

Reviewers comments:

Dr. Giovani dos Santos Cunha (Reviewer 1)

*General Comments*

*The manuscript provides information on external validation of VO2max prediction models based on recreational and elite endurance athletes. Overall, the study is well-conducted, methodologically robust, and provides relevant information for the area of sports and health sciences. My concern about the study is mainly related to the introduction, methodology, and discussion sections, which could be edited. Please, see in the specific comments.*

*Specific Comments*

*Personal suggestion: I believe the main message of the study is that direct VO2max determination (CPET plus ergospirometry) cannot be replaced or interchangeable by predictive equations for endurance athletes based on their own results 0.30 < R2 < 0.65. If the authors agree with me, please make this message clearer in the discussion and conclusion of the study. Additionally, little is discussed about the repercussions of using predictive equations with low to moderate accuracy for the contexts of sports & performance diagnostics, clinical practice, applicability of prediction equation, training prescription and follow-up .*

Response: Thank you for the suggestion, we have added this information to the introduction section.

*Abstract:*

*What is the criterion used to define models with the highest accuracy?*

Response: We added information: “by assessing the transferability of the currently available prediction models based on their R2, calibration-in-the-large, and calibration slope”

*Please, include the minimum and maximum values for underestimated and overestimated that can be obtained for indirect VO2max.*

Response: Due to the limited capacity of the abstract, we are not able to provide as much detail for each of the validated equations. These details are given in the results section and the supplement

*Introduction: The introduction does not convince the reader of the importance of direct determination of VO2max compared to indirect determination through equations. Additionally, the authors could include epidemiological data to strengthen the argument for the importance of VO2max for the health of endurance athletes. Please make it clear in the introduction what is the main focus and secondary outcomes of the study. In addition, to include the advantages and disadvantages of each of the ways of determining VO2max for the contexts of sports & performance diagnostics, clinical practice, applicability of prediction equation, training prescription and follow-up .*

Response: Thank you very much for your insightful analysis of the content. We have amended the content in line with your suggestions

*Lines 18-19: Please, describe more variables that can influence the variability of VO2max data in endurance athletes.*

Response: We added additional variables as suggested

*Lines 33-34: Please, deepen this information, including epidemiological data, if possible, on CVDs in endurance athletes. Please, include information here about the importance of directly measured VO2max can be used as a predictive cardiometabolic risk factor. In addition, there is little in the introduction about the limitations or harm that the indirect determination of VO2max may have in relation to clinical evaluation. Please, present the risks and benefits of direct and indirect determination (prediction equations) of the VO2max data.*

Response: We have added more detailed information including epidemiology and clinical evaluation

*Line 55: There is little information about why endurance athletes were chosen as a sample for the study* .

Response: We have added information in this regard

*Methodology:*

*Lines 79-80: Please make it clear whether data collection was performed with a breath-by-breath acquisition system and a 10-seconds filter was used for data analysis. Note that this information can lead to some confusion as to how VO2max was defined as described in line 104 “Maximal oxygen uptake (VO2max) was defined as an averaged maximum oxygen uptake during the 15-s period at the end of the CPET’. Could I think that only 1 or 2 VO2 points were analyzed for the average of VO2max? Please clarify .K*

Response: We have corrected this section. Measurements were taken for each breath and averaging was done at 15-second intervals. The 10-second values were entered by mistake. Many thanks for finding this inaccuracy in the text

*Suggestion: Please, insert the 13 equations of selected predictions models to establish pVO2max in methodology section*.

Response: Thank you for your suggestion but we do not want to duplicate content. A detailed table describing all included equations can be found in the manuscript supplement. In the main body of the paper, such a table would take up a considerable amount of space. Bibliographic data for the included designs are also included in the supplement

*Statistical Analysis: Please, describe the criterion used to classify low to moderate variability in VO2max. What is the criterion used to classify accuracy (low-moderate-high)? Please, describe*

Response: The criterion for determining the accuracy of the models was R2 and RMSE, as described in lines 173-183. We did not use cut-off points for these parameters, as there is no explicit definition adopted here as in, for example, correlation coefficients or effect measures.

*Discussion: The authors could better highlight the main findings of the study. In addition, authors should focus the discussion section on possible explanation of their main results of the present study.*

Suggestion: The authors could organize the discussion as follows: Describe the consequences (risks and benefits) of using VO2max prediction models with low to moderate accuracy (underestimation and overestimation values) in the following contexts: (1) Sports & Performance Diagnostics; (2) Clinical Practice; (3) Endurance Training Prescription and Follow-Up.

Response: We have made amendments

*Reviewer #2: I don't understand very well this study. If you check the correlation between a previously validated formula in very specific conditions with actual values in another different setting, it is obvious that the results will be quite different. The authors should guarantee that the comparisons are made with the same protocols (step duration, starting intensity, exhaustion criteria, etc.) and populations (age, sex, sport, training level, etc.) between the current and previous validation studies*

Response: We have added appropriate explanations in the text, mainly in the introduction of the manuscript and methodology regarding the need for external validation and differences in protocols and VO2max values obtained. By following the guidelines for validation studies (such as the TRIPOD statement), we obtained a cross-sectional analysis of the available equations, which enabled us to reach appropriate conclusions.

*The figures are not legible.*

Response: This is most likely a matter of converting the document in the editorial system for review purposes. The uploaded charts are in the correct high resolution and size. In the PLOS submissions system, they are presented on A4 size, each on a separate page. In the top right corner of the manuscript there is a hyperlink to download the TIFF version. A higher resolution version is then downloaded

*VO2max can be tested on the field in a sport setting. An example:*

*https://pubmed.ncbi.nlm.nih.gov/24936896/*

Response: Thank you very much for your valuable publication, we have completed the information

**Once more, we would like to thank the Reviewers for their important comments.**
