# Supplementary material for: External validation of VO2max prediction models based on recreational and elite endurance athletes
Source: PLoS One. 2023 Jan 25;18(1):e0280897. doi: 10.1371/journal.pone.0280897 (PMC9876283; doi:10.1371/journal.pone.0280897)
Supplement: Attachment — Submitted filename: response to Reviewer 2.docx [file pone.0280897.s007.docx]

***Dear Editor,***

***Dear Reviewers,***

*Thank you very much for the thorough analysis of our manuscript, for your valuable and helpful comments and for giving us the opportunity to revise and improve our submission. We hope that our replies and explanations, as well as the amendments to the manuscript, fully address your concerns. We keep the change tracking. In the following, please find our answers to your comments.*

*Reviewer #2: Please, elaborate better on the error measures reported, the potential sources of these errors, and the differences among protocols and formulae in the discussion section.*

**Response: We have added a new section to the discussion**

*Once more, we would like to thank the Reviewer for important comments and for input on our report. We believe that all remaining concerns are now fully addressed.*

*With regards*

*Szczepan Wiecha, PhD*
